# A 4 Year Human, Randomized, Radiographic Study of Scalloped versus Non-Scalloped Cemented Implants

**DOI:** 10.3390/ma13092190

**Published:** 2020-05-10

**Authors:** Bruna Sinjari, Gianmaria D’Addazio, Manlio Santilli, Barbara D’Avanzo, Imena Rexhepi, Antonio Scarano, Tonino Traini, Maurizio Piattelli, Sergio Caputi

**Affiliations:** Department of Medical, Oral and Biotechnological Sciences, University “G. d’Annunzio” of Chieti-Pescara, 66100 Chieti, Italy; gianmariad@gmail.com (G.D.); santilliman@gmail.com (M.S.); barbaradavanzo@hotmail.it (B.D.); imena.rexhepi@gmail.com (I.R.); ascarano@unich.it (A.S.); t.traini@unich.it (T.T.); mpiattel@unich.it (M.P.); scaputi@unich.it (S.C.)

**Keywords:** dental implants, bone resorption, marginal bone loss, scalloped implants, radiographic analysis

## Abstract

Marginal bone loss (MBL) is a key factor in long-term implant success rate. Among the different factors that influence MBL, it is the different implant shoulder designs, such as scalloped or non-scalloped, which have been widely studied on screw retained but not on cemented retained implants. Thus, the aim of the present study was to evaluate the MBL around scalloped and non-scalloped cemented retained dental implants after 4 years of loading, in humans. A total of 15 patients were enrolled in the present study. A radiographic and clinical examination was performed after implant placement (T0) and after 4 years from it (T1). The results demonstrated a differential MBL (T1-T0) of 2.436 ± 1.103 mm and 1.923 ± 1.021 mm, respectively for test (scalloped) and control (non-scalloped) groups with a statistically significant difference between them. On the other hand, no statistically significant differences were found between the groups in terms of prosthetic complication and abutment decementation, whilst ceramic crowns chipping was shown in both groups. In conclusion, the use of a scalloped platform did not provide better results on the maintenance of MBL after 4 years follow-up. In this study, this probably was determined by multiple factors, among which was the subcrestal insertion of scalloped implants.

## 1. Introduction

The preservation of the crestal bone has become one of the main objectives in oral implantology [1,2]. According to Albrektsson’s criteria, crestal bone loss around an implant occurs conspicuously at the time of implant insertion and up to 6 months, and to a smaller extent, in the following years after load [3]. However, if this loss progresses uncontrollably, the biomechanical anchoring of the implant-supported prostheses can be drastically compromised [4,5]. In fact, even if the reaching of osseointegration of an implant is essential for gaining success, it does not necessarily reveal that this system will preserve its integrity during the patient’s life because a lot of aspects can influence the kinetics of mineralized tissue [6,7]. It is in fact a common consent that the preservation of “long-term” hard tissue around the dental implants is one of the most important aspects in the realization of an implant restoration, and that the progressive loss of bone substance drastically reduces the chances of survival of the dental implants under occlusal load [8,9,10]. It is the synergy of different conditions that mediates the progressive loss of the mineralized tissue around the dental implants, therefore the loss of peri-implant bone is of multifactorial origin [6,11]. Among the causes of this phenomenon there are: trauma during the surgical procedure; exposure of the implant during soft tissue healing; infections/inflammations during the healing period; bacterial colonization in the fixture-abutment gap with consequent inflammation of the crestal tissues; excessive occlusal forces; early loading in the presence of a biomechanically inadequate bone-biomaterial interface; incongruous macrostructure of the implant etc., [1,12,13,14]. All of these variables largely decrease the purely technical factor as the cause of crestal bone loss while other causes can be assessed/avoided through the use of implants designed following specific engineering concepts [1,14]. Regarding this, it is important to underline how surface topographies are designed to respond precisely to cell adhesion and positively stimulate cell selectivity [7]. Alterations in surface morphology, for example, caused by professional oral hygiene maneuvers, can negatively alter the implant surface [15]. This happens not only at the bone level but also at the soft tissue level, where the surface morphology can influence the cellular response [16]. On the other hand, many researchers have shown how the implant macro and micro-geometry can play an essential function in the long-term conservation of the bone crest [17,18]. A recent systematic review and meta-analysis concluded that changing implant shoulder positions, shape or orientations (scalloped, sloped, and one piece) offers no advantage when compared to biphasic standard flat implants, with sufficient scientific evidence. Moreover, marginal bone resorption appears to be affected by the implant neck design, meanwhile patient satisfaction and aesthetics results seem to be not involved [18]. Another review demonstrated that restoration with a scalloped implant–abutment connection revealed significantly more peri-implant bone resorption compared to implants with a standard implant–abutment shape [19]. A further key factor is linked to the presence of microgaps in biphasic implant rehabilitations. The latter is defined as the microscopic space between fixture and prosthetic components. This space allows bacterial micro-infiltration influencing the long-term survival of implant-supported rehabilitations [2]. Despite the numerous studies performed worldwide to the authors best knowledge, there are no studies where marginal bone loss (MBL) has been compared around dental implants where the only variable was the different implant shoulder (scalloped versus non-scalloped) in a cemented implant system.

Thus, the aim of the present study was to radiographically analyze the MBL on scalloped and non-scalloped cemented implants after 4 years follow-up.

## 2. Materials and Methods

### 2.1. Study Design

This randomized controlled single center study was designed according to the Declaration of Helsinki protocol. The study was approved on 19/12/2007 by the Inter Institutional Ethics Committee of University of Chieti-Pescara, Chieti, Italy—committee report nr:ME1. All patients provided a scripted informed consent to the study recruiting and surgical-prosthetic treatment. Each patient provided only 2 implant sites for the study, one scalloped (test group) and one non-scalloped (control group). These sites were determined at the time of recruitment by the clinician and selected based on the greatest anatomical similarity. The implants were supported exclusively by single crowns. The implants were evaluated after 4 years from implant placement.

The null hypothesis was that there were no differences between the two types of implant designs on MBL after 4 years follow-up. The primary outcome was the mean of MBL in single implant-supported restorations of scalloped and non-scalloped implants. All implants were restored by a single metal ceramic implant supported crown. MBL was used to estimate the number of patients needed to be randomized. Moreover, prosthetic complications such as abutment and/or crown decementation, crown fracture or ceramic chipping at 4 years follow-up were recorded.

### 2.2. Patient Selection

Fifteen patients who needed two single implant supported restorations were incorporated in the present study. The patients enrolled were 10 males and 5 females, aged between 31 and 78 years old with a mean age of 60 years, each of whom must receive two implants, respectively scalloped and non-scalloped. After taking into consideration the difference in terms of MBL between two groups, where the different fixture-implant was evaluated [20], a sample size of 13 patients per group was calculated to have at the follow-up a minimum difference of MBL. Specifically, the study of Pozzi et al. [20] showed three-year post-loading results, comparing implants with different prosthetic interfaces and designs in partially posterior edentulous mandibles. In their study, the MBL mean was 0.67 ± 0.39 mm and 1.24 ± 0.47 mm for the two groups of implants taken into consideration. In fact, a statistically significant difference between the groups was shown. The value of α was determined at 0.05, while the power of the test was 0.95. For the calculation, the Pass 3 software was used and specifically the Two-Sample *T*-Tests taking Equal Variance. The sample size was increased to avoid patient losses at follow-up, which would invalidate the test. Then, 15 patients were included to compensate a possible drop-out.

The patients enrolled were treated in the Outpatient Department of Medical, Oral and Biotechnological Sciences of the University “G. d’Annunzio” of Chieti-Pescara, Italy. The inclusion criteria were as follows: patients between 18 and 75 years old of both sexes; partially edentulous who needed at least 2 adjacent single implant rehabilitations on both the upper and lower jaws; patients who had a residual crest of 5.3 mm wide and at least 10 mm long to allow implant insertion; each patient contributed with only two single implants in this study; a 1 mm wall thickness of the vestibular and palatal plate was necessary, therefore, considering the implant diameter, a residual ridge of 8 mm wide was required.

Meanwhile, the exclusion criteria were: general contraindications to implant surgery; smoking more than 10 cigarettes per day; patients irradiated to the head or neck less than 2 years; patients undergoing chemotherapy for less than 1 year; patients with uncontrolled diabetes; pregnant and lactating women; post-extraction sites with acute or purulent infections; post-extraction sites with non-intact walls; post-extraction sites with implant-bone gap > 2 mm; serious disorders of clotting, patients with uncontrolled systemic or metabolic disease; drug and alcohol abuse; patients with periodontal disease (evaluated and recorded by plaque score (PS) and bleeding score (BOP) on four surfaces on each tooth > 25%) [21]; patients with poor oral hygiene and motivation; patients treated with bone augmentation surgical techniques < 6 months; patients participating in other clinical trials, in case they interfere with the application of the present protocol.

**Figure 1:**
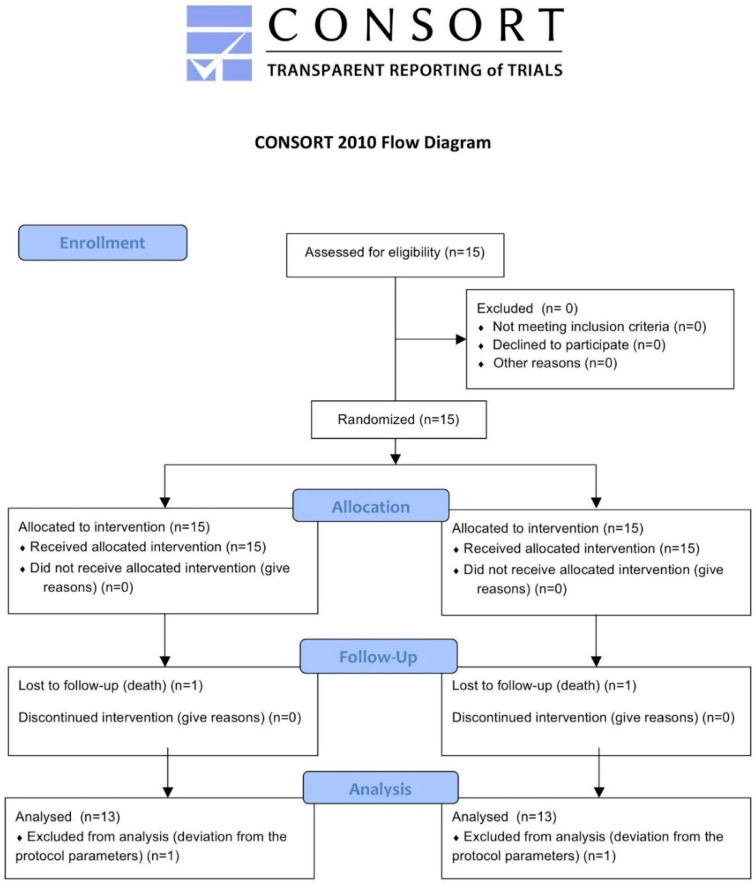
CONSORT 2010 Flow Diagram.

This article was written following the CONSORT statement in order to display the progress of all participants through the trial, as shown in Figure 1 [22].

### 2.3. Randomization

Patients were allocated into test group (scalloped) and control group (non-scalloped) as indicated by the randomization chart. A computer generating random numbers was used for the randomization and centralized with sequentially sealed opaque envelopes provided by the study adviser. Procedures to keep the allocation of the implant hidden from operators (Allocation Concealment) were performed. In fact, the operator revealed the sealed envelope containing the randomized group only after completing the preparation of the two implant sites and only just before inserting the implant.

### 2.4. Surgical and Prosthetic Treatment

**Figure 2:**
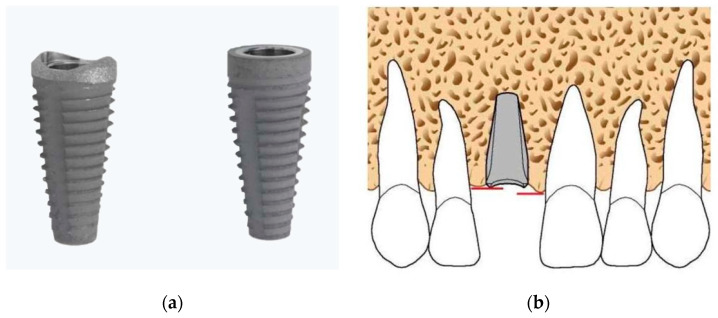
(**a**) Image of both bone system dental implant fixtures (left: scalloped shoulder design, and right: non-scalloped or flat shoulder implant); (**b**) Implant positioning scheme of scalloped implants where the upper approximal margin of the implant neck was positioned at the level of the lowest bone peak indicated by red lines.

During the first visit, all subjects were clinically examined through radiographs and gingival indexes, such as, PS and BOP, and were then scheduled for surgery procedures. All implants (Bone System s.r.l, scalloped and non-scalloped, Milan, Italy) were inserted (T0) by two skilled operators (M.P. and A.S.), who followed a two-stage protocol and placed them according to the manufacturer’s instructions. The implants used in this study have general characteristics not dissimilar to the standard implants. A root-shaped implant, whose main feature is given by the design of the implant platform, consisting of a scalloped coronal part, was used. The sandblasting and acidification treatment extend over the entire implant surface, also affecting the festooned portion and the implant platform. All implants had a diameter of 5.3 mm and lengths of 10 and 12 mm as shown in Figure 2.

All the patients were subject to professional oral hygiene 1 week before, and a mouth rinse of chlorhexidine digluconate solution 0.2% for 2 min immediately before surgery to reduce the bacterial load. Local anesthesia was given with Articaine (Ubistesin 4%—Espe Dental AG Seefeld, D-82229 Seefeld, Germany) associated with epinephrine (1:100,000). The incision of the flap was carried out to not damage the papillae of the adjacent teeth, if any, through the curvilinear incision described by Sclar [23]. The insertion axis of the implant was performed to not affect the vestibular bone wall.

In case of 2 contiguous implant positionings, a minimum inter-implant distance of 3 mm was maintained meanwhile the distance between the implant and the adjacent tooth was at least 2 mm.

The implants were positioned in the alveolus using a handpiece combined with a controlled torque handpiece and then, if necessary, inserted manually with the ratchet as indicated by the manufacturer. The upper approximal margin of the implant neck must be positioned at the level of the lowest bone peak (Figure 2). The closure of the flap above the implant was primarily intended with Vicryl 4.0 (FS-2, Ethicon, Somerville, MA, USA). Amoxicillin with clavulanic acid was administered, with a dose of 2 g/day for 6 days (Augmentin; Glaxo-Smithkline Beecham, Brentford, UK). The postoperative pain was controlled with NSAIDs, a cold/soft diet was suggested for 2 weeks, together with appropriate oral hygiene. The sutures were removed 7 days after implant insertion. The surgical protocol was performed as already described [24].

Three months after insertion, the implants were uncovered and a standard healing transmucosal collar was applied. After 21 ± 7 days, the impression was taken with the specific transfers, and then, sent to the dental laboratory, which prepared the final customized collars and parallelized titanium abutments including the temporary element. The definitive gold ceramic crown was applied within the fifth month from implant placement.

### 2.5. Data Handling and Radiographic Analysis of Marginal Bone Level Changes.

The implant’s success was assessed according to the radiographic and clinical criteria of Papaspyridakos et al. 2012 [25]: (1) absence of implant mobility; (2) absence of pain; (3) absence of recurrent peri-implant infection; and (4) absence of a continuous radiolucency around the implant. Data were collected in the specific patient’s case report forms.

The peri-implant gingival index was also recorded. Moreover, during each visit, possible adverse events and prosthetic complications were collected. In order to evaluate radiographic change of the peri-implant bone, intraoral radiograph, applying the parallel ray technique, was realize after implant surgery. To evaluate the MBL, intraoral analogic Rx was performed during each stage and processed on a digital software, since the method was considered of high precision for evaluations [26] with a precision less than 0.1 mm as previously reported [2]. The mean value between mesial and distal region was used as the primary outcome measure for this study.

The commercially available Rinn film holders, used for intraoral radiographs applying the parallel X-ray technique, were customized using a silicone key for the exact reposition in every subject, in order to obtain a highly reproducible and faithful radiograph. Furthermore, during the first radiography, kilovolts, milliampere, and seconds were registered and used in all the stages to obtain the same images. Radiographs were repeated at implant placement (T0), and at 4 years follow-up (T1).

The values obtained were expressed in terms of average and standard deviation (mean ± SD) on a millimeter scale.

For the radiographic measurements, we used a protocol already described previously in the literature [2], where a computer-assisted calibration was used to guarantee a correct measurement. Briefly, to calibrate the software the known implant diameter and length were measured and inserted then the distance from the implant shoulder and the first bone implant contact was measured both horizontally and vertically. Consequently, the mean value was recorded in the software. The measurements were performed for the mesial and distal side in both study implants. The measurements were repeated for each study time point.

In every radiograph, distance from the top of the fixture (implant shoulder) to the first bone to implant contact, both mesial and distal sides were measured. The mean value between mesial and distal region was calculated for the data analysis. A negative value (−) was given in those cases where the MBL was below the implant shoulder, taking into consideration that both implants were inserted in T0 in a subcrestal position. In addition, the known implants’ lengths and diameters were measured to guarantee a correct measurement, even if the implant was slightly angulated on the radiograph.

Based on this ratio, a computer-assisted calibration was performed and linear measurements of MBL were taken using ImageJ 1.48 v.; Bethesda, MD, USA.

The software was calibrated for each individual image using the known distance of the implant diameter at the neck (5.3 mm).

### 2.6. Statistical Analysis

Statistical analysis was performed using a computerized statistical software; specifically, SPSS (V. 24-0-IBM Corp., Armonk, NY, USA) was used.

The unit of analysis was the implant, not the number of patients. Then, singular patients were treated twice (for test group and control group implants).

The data were analyzed with descriptive statistics (Kolmogorov–Smirnov test) to evaluate whether they had a normal distribution. The differences between groups for peri-implant bone levels were compared using the paired *t*-test. Moreover, a Pearson correlation coefficient (R) was used to investigate the correlation between MBL and the different possible influencing factors: age, sex and implant position. All statistical comparisons were conducted with a significance level of 0.05.

## 3. Results

This study highlighted the late failure of an implant, which occurred in a period of about 10 months after implant placement, with loss of osseointegration. A few months later, a new implant was inserted then perfectly osseointegrated. All the remaining implants were perfectly osseointegrated and clinically stable. No implant fractures occurred, but in one patient, the decementation of the temporary prosthesis was recorded and immediately repositioned. All patients reported good periodontal health, with periodontal bleeding on probing and plaque score values under < 25%.

The main patient demographic characteristics as well as MBL at T0, T1 and the differential values are described in Table 1.

After implant insertion, only one patient experienced paresthesia of the lower hemilip, which was resolved completely after one month. A total of 13 out of 15 patients completed the study. In addition, two patients left the protocol, the respective causes of these losses were: death in one case; and in the other case, deviation from the protocol parameters described above, as the patient did not show up for several fixed appointments and returned after a long time with several lost dental elements. In this case, the prosthesis was not delivered due to the necessary change of the treatment plan, following the new clinical situation.

**Figure 3:**
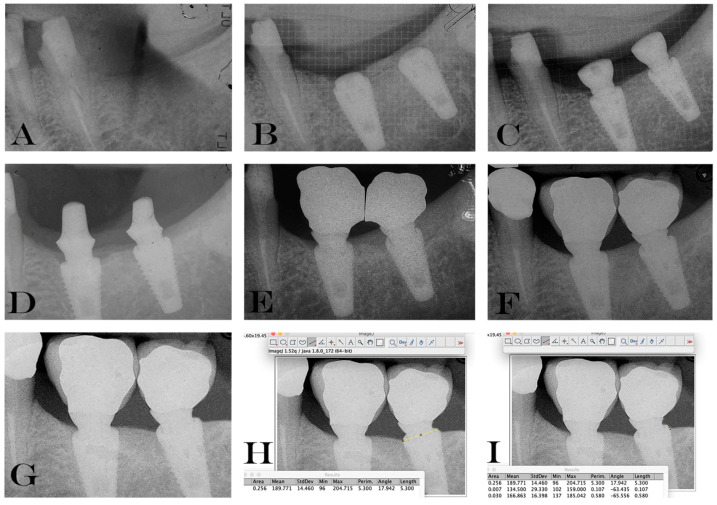
Periapical radiographs showing all the phases of the study (**A**–**G**): (**A**) pre-operative periapical radiography; (**B**) immediately after implant placement; (**C**) at the second surgical stage; (**D**) cementation of final abutments; (**E**) crown structure test; (**F**) delivery of the permanents metal-ceramic crowns; (**G**) radiograph taken 4 years after implant insertion. (**H**–**I**) demonstration of the MBL measurements with computer-assisted calibration to guarantee a correct measurement also in cases where the radiograph was slightly angulated.

**Figure 4:**
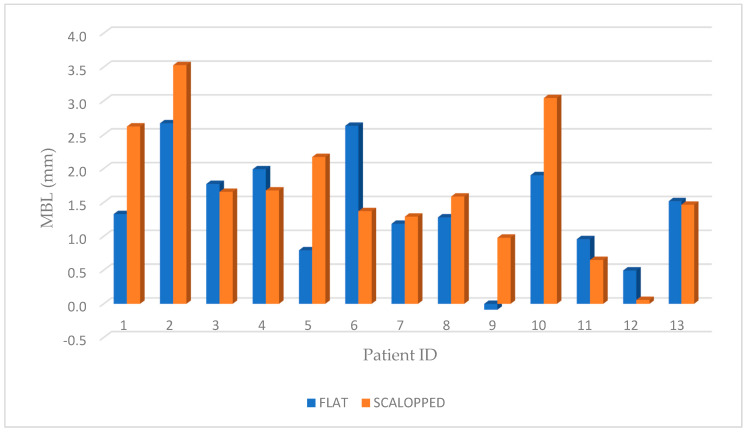
Graphic representation of marginal bone level of both groups at T0, where no statistically significant difference was shown between the test (scalloped) and control (flat) groups (*P* = 0.2325).

**Figure 5:**
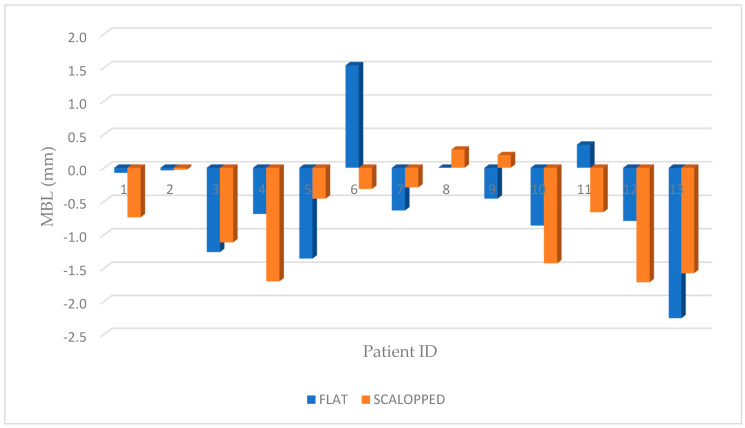
Graphic representation of MBL of both groups, for each patient, at T1 where no statistically significant difference was shown between the test (scalloped) and control (flat) groups (*P* = 0.333).

Marginal bone resorption measurements were carried out at the time of implant insertion and at the end of 4 years follow-up, the values obtained are expressed in terms of average and standard deviation (mean ± SD) on a millimeter scale as shown in an explanatory case in Figure 3. All implants were inserted with the upper margin of the fixture positioned at the level of the lowest bone peak as demonstrated on Figure 2b. Thus, the measurements made at T0 showed a mean marginal bone level of 1.699 ± 0.95 mm in test group and 1.418 ± 0.79 mm in control group. No statistically significant differences were found between the two groups at T0 (*P* = 0.2325) (Figure 4). MBL measured at T1 from the crestal bone level was −0.73 ± 0.70 mm in test group and −0.50 ± 0.92 mm in control group. Additionally, in T1, no statistically significant differences were found between the two groups (*P* = 0.333) (Figure 5).

However, at T1, the results demonstrated a differential MBL, from starting point after 4 years, of 2.436 ± 1.103 mm and 1.923 ± 1.021 mm, respectively for test and control groups.

**Figure 6:**
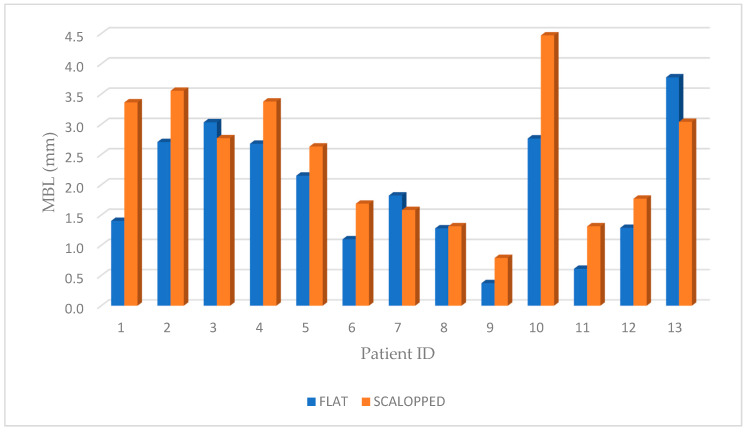
Graphic representation of MBL of both groups at T1, after 4 years follow-up (T1-T0), where a statistically significant difference was shown between the test (scalloped) and control (flat) groups (*P* = 0.02).

**Figure 7:**
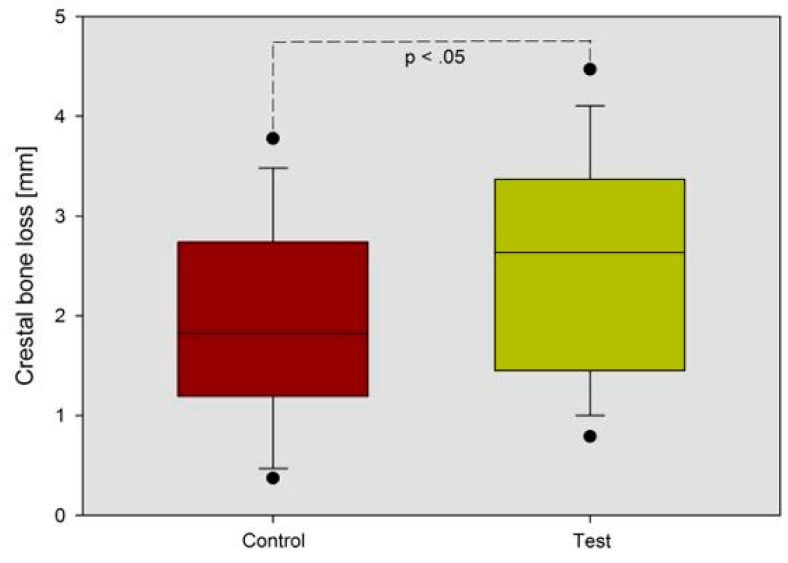
Box plot graphic representation of MBL of both groups after 4 years follow-up, where a statistically significant difference is shown *p* < 0.05 between the test and control groups (*P* = 0.02).

The paired *t*-test (*P* = 0.02) demonstrated a statistically significant difference between the two groups (Figure 6; Figure 7). Moreover, for “non-scalloped” implants, the minimum value reported was 0.373 mm, and the maximum value reported was 3.777 mm, whilst, for “scalloped” implants the minimum and maximum value reported were 0.791 and 3.555 mm, respectively. The relationship between the different values recorded at T0, T1 and in the differential (T1-T0) are shown in detail in Figure 4, Figure 5 and Figure 6. As mentioned, the paired *t*-test did not demonstrate statistically significant differences in the samples at T0 and T1; instead, it was in the differential. Analyzing two implants per patient, almost all couples, excluding patients 3, 7 and 13, exhibit significantly greater resorption in the test sample. Meanwhile, in patients 3, 7 and 13, the control sample shows a greater resorption differential but with minimal differences (Figure 4, Figure 5 and Figure 6).

To better understand the data, a possible correlation with sex and implant position was also investigated. The correlation between the differential and increasing age showed R = −0.29 value in the control group, demonstrating a negative correlation but without any statistical significance (*P* = 0.33). On the contrary, in the test group, the correlation was positive but always with non-statistically significant values (R = 0.25, *P* = 0.41) also in this case. No MBL differences were observed regarding sex (Table 2); however, implants placed in all women showed lower differential MBL with no statistically significant difference (*P* = 0.47).

Regarding implant site position, no difference was shown between groups for this cause of the adjacent implant insertion site. In fact, in all patients, the implants were placed in equivalent anatomical positions to avoid bias related to the anatomical site. Correlation between maxillary implant position and MBL has been studied but no statistically significant differences have been detected (R = 0.53, *P* = 0.06, control group), (R = 0.28, *P* = 0.33, test group). Implants placed in maxillary bone showed higher differential MBL with no statistically significant difference (*P* = 0.057). Finally, the mean MBL for the cohort with all the details is illustrated in Table 2. 

Moreover, no statistically significant differences were found between the two groups in terms of prosthetic complications. These data both with decementation and chipping rates have been presented in Table 3. No abutment or permanent crown decementation was observed in both groups. On the other hand, regarding ceramic chipping, it occurred in both groups with a percentage of 23.07% (three cases) and for 15.38% (two cases) in the test and control group, respectively. Meanwhile, a total ceramic chipping rate of 19.23% after 4 years of function was found.

## 4. Discussion

The null hypothesis of this study was rejected, demonstrating that a difference in terms of MBL was detected between groups. The results of this study highlighted how the use of implants with a scalloped platform does not bring additional benefits regarding MBL, comparing it with the results obtained by the use of flat implant platforms. On the contrary, better results were shown at 4 years follow-up with the flat design. In the literature, preliminary results regarding the use of a scalloped platform seem to be unfavorable to the maintenance of peri-implant bone tissue. Our data are in fact in agreement with those of Park et al. in 2010 [27], which used 4 types of implants with a festooned collar, different from each other only for microstructure, highlighting that the use of a festooned collar does not prevent crestal bone resorption. In the same study, however, it is noted that the use of an implant with a festooned collar associated with a micro-threading was able to ensure less extensive bone resorption [27]. Moreover, a main difference exists between the two studies, as their study was performed in dogs, meanwhile ours was performed in humans.

Moreover, Bradley in 2007 compared monolithic and biphasic implants with festooned collars, evaluating the differences in marginal bone resorption [28]. They showed that an enhanced interproximal tissue preservation from scalloped implant designs may lead to more predictable esthetic dental implant restorations in the anterior maxilla. Considering only the biphasic implants, the follow-up period had an average of 15 months and the identified crestal bone resorption was approximately 2 mm [28]. On the contrary, in the present study, a follow-up period of 48 months was analyzed, therefore the higher resorption values appear justified.

Furthermore, we also found consistency with the data published by Kan et al. in 2007 [29], who concluded by saying that the crestal bone tissue was not preserved at the original level in sites with scalloped platform implants. Finally, a direct correspondence of the present results with those of Nowzari et al. in 2006 was found [30]. In fact, they concluded by saying that the use of implants with a scalloped platform determines a greater bone resorption than implants with a flat platform.

However, there are studies that demonstrate the real effectiveness in the use of implants with festooned platforms. Khatami et al. in 2006 [31] declared how the clinical and radiographic results support the theory that implants with a scalloped platform preserve the maintenance of the crestal bone, consequently guaranteeing the support of the interdental papilla.

In addition, more recently Starch-Jensen et al. in 2017, have carried out a systematic review of the literature [19]. The goal was precisely to identify differences between the various outcomes after treatment with flat or scalloped design systems. In this paper only 3 studies fulfilled the inclusion criteria. The included studies always reported a high implant survival rate and a lower MBL in flat design [32,33,34]. The included studies showed a different follow-up period: 12 [32], 36 [33] and 60 [34] months, respectively. In our case, a 48-month period was analyzed. Authors concluded that at 5 years, implants with a scalloped design had significantly higher bleeding, gingival and probing depth scores than implants with a flat design [19]. However, none of the included studies used a cemented implant–abutment connection, as here reported [19]. It should be considered that our study is the one, to the authors’ best knowledge, that treats the MBL around cemented dental implants. All the above-mentioned studies treat screw retained abutments. In vivo and in vitro studies suggested that the internal implant connection of cemented retained abutment allows a greater degree of isolation from the peri-implant sulcus than in screw retained, and therefore, guarantees greater benefits in terms of bacterial colonization and microgap reduction [35,36].

Over the years, some studies evaluated different aspects related to this type of connection. Assenza et al. in 2006 [36] investigated the effect of the cemented retained abutment and its possible decementation on peri-implant soft tissues. The same authors in 2012, on the other hand, evaluated that bacterial infiltrate in the microgap in different implant–abutment connections, demonstrating a better performance of the cemented connection [35].

Being that marginal bone loss is a multifactorial phenomenon, where plaque accumulation plays a key role, the cemented implants could positively affect the bone loss. On the other hand, it has been reported that the presence of the cement in the peri-implant sulcus may lead to peri-implantitis and therefore implant failure [37]. Thus, it is of relevant importance to perfectly remove the cement from the peri-implant sulcular once the abutment is cemented.

However, the authors published a recent paper where no statistical differences were shown between screw retained and cemented implants after 10 years of follow-up [4].

Thus, the importance of these results is in accordance with the trend showed in screw retained abutments connection type. It is therefore necessary to carefully read the results of this study, which highlight a better maintenance of the crestal bone in favor of implants with a flat platform on cemented abutments implants. Analyzing the individual cases, only in 3 out of 13 patients there was a better maintenance of the crestal bone on the implants with a scalloped platform. However, a more careful analysis has shown that in these patients, the level of the crestal bone at the baseline by test and control was almost similar (the maximum difference between test and control was 0.1 mm). Moreover, in 6 out of 13 patients, a more subcrestal positioning of the test implants was found, justifying the greater bone resorption in these cases. In the remaining 4 out of 13 patients, although there is a more subcrestal positioning for the control implants, the latter nonetheless showed less bone resorption [31]. The positioning of the implant above or below the crest acquires importance in relation to the formation of the biological width, which forms in an apical direction, starting from the fixture–abutment junction. In fact, it follows that the more subcrestal the implant is positioned, the more apical the formation of the biological width, which in turn, needs space for the formation of the epithelium and its sub-epithelial connective portion. The more apical the position of the implant margin will be, the more it will be that of the bone margin [38,39]. The positioning above or below the crest of an implant is a fundamental determinant for the maintenance of the crestal bone, as has been described in studies on animals and on humans [40,41,42].

The positioning of a subcrestal implant is related to a greater reabsorption of bone not only for the necessary formation of biological width but also because the preparation of a deeper implant site is linked to a greater traumatized bone surface, and therefore, to greater vascular damage [42]. Taking into account that the implants used had a 5.3 mm diameter, the greater diameter extension led to further removal of bone tissue. The interruption of part of the vascular network of the bone tissue at the implant site is the cause of a reduced blood supply in the peri-implant areas and this probably could partly explain the reason for a greater bone resorption.

As pointed out, the multifactorial of the crestal bone resorption places a limit on the results obtained by evaluating the macro-morphology of the implant platform alone.

In any case, the results presented demonstrate an acceptable level of marginal bone resorption in both the methods used, but the variable examined seems to influence the resorption over the years. A low rate of prosthetic complication was also showed. Papaspyridakos et al. in 2019 demonstrated a rate of “prosthesis free of technical complications” of 56.4% [43]. Instead, results here presented showed 80.77%, at 4 years follow-up. However, a total ceramic chipping rate of 19.23% after 4 years of function was shown on both groups with a percentage of 23.07% (3 cases) in test and of 15.38% (2 cases) in the control group. On the other hand, no other prosthetic complications were observed in both groups in terms of abutment or crown decementation during the study.

However, it has to be highlighted that the festooning of the implant neck could be important from an aesthetic point of view, since the maintenance of the crestal bone guarantees adequate support for the soft tissues, and therefore, the formation of the interdental papilla [44,45,46]. The stability of the soft tissues could prevent the formation of interdental black triangles and the uncovering of the metal edges around the implant neck, both causes of unacceptable imperfections, capable of affecting the result of the therapy [47,48]. This could be taken into consideration as the objective of the implant-supported rehabilitation is not only to provide patient with correct function but also with a correct aesthetic.

One of the main limitations of the present study is the small sample size, although based on a previous paper for determining it. Thus, clinical trials with a bigger sample size must be performed to validate the results of the present study and moreover, to better understand the MBL around cemented implants.

## 5. Conclusions

In summary, in this study, the use of a scalloped platform did not provide better results on the maintenance of the crestal bone after 4 years follow-up. This, probably, is determined by multiple factors, among which the subcrestal insertion of festooned implants stands out. The preparation of the implant bed results in reduced vascular function as the surgical hole is deeper. In any case, all implants, whether with scalloped or non-scalloped platforms, after 4 years follow-up, were well osseointegrated. On the other hand, prosthetic complications such as crown chipping were shown in both groups, with a higher presence in the test group (3 cases) compared to the control group (2 cases). Further studies, with the same study design but with a greater sample size, are needed to clarify whether the scalloped implant platform can indisputably contribute to the maintenance of peri-implant soft tissues.

## Figures and Tables

**Table 1:** Table shows demographic characteristics, site, bone density, insertion torque and radiographic MBL measurements at different time points of all the patients enrolled in the study. The minus (−) indicates that the quantity of bone was lost below the implant shoulder (please see Figure 2). A difference was determined between the values obtained of T1-T0 to have the amount of bone resorption at 4 years follow-up.

Id	Age	Sex	Group	Site	Bone Density	Final Torque NCM	T0 Rx (mm)	T1 Rx (mm)	Rx T1-T0
1A	78	M	CONTROL	46	Normal	45	1.329	−0.076	**1.404**
1B	78	M	TEST	47	Dense	35	2.622	−0.741	**3.363**
2A	67	F	CONTROL	16	Poor	25	2.669	−0.039	**2.708**
2B	66	F	TEST	14	Normal	40	3.528	−0.028	**3.555**
3A	48	F	CONTROL	25	Poor	30	1.773	−1.261	**3.034**
3B	47	F	TEST	24	Dense	45	1.656	−1.115	**2.771**
4A	67	M	CONTROL	24	Normal	40	1.990	−0.690	**2.680**
4B	68	M	TEST	26	Normal	45	1.677	−1.700	**3.377**
5A	76	M	CONTROL	35	Dense	50	0.794	−1.358	**2.152**
5B	77	M	TEST	36	Normal	35	2.171	−0.462	**2.633**
6A	59	F	CONTROL	37	Normal	30	2.633	1.532	**1.101**
6B	60	F	TEST	46	Dense	40	1.371	−0.317	**1.688**
7A	45	M	CONTROL	25	Poor	25	1.185	−0.638	**1.823**
7B	44	M	TEST	24	Normal	30	1.290	−0.294	**1.584**
8A	68	M	CONTROL	26	Normal	25	1.280	0.000	**1.280**
8B	69	M	TEST	27	Poor	30	1.587	0.272	**1.315**
9A	56	M	CONTROL	46	Dense	50	−0.089	−0.462	**0.374**
9B	57	M	TEST	47	Normal	55	0.979	0.188	**0.791**
10A	73	M	CONTROL	27	Normal	40	1.902	−0.864	**2.766**
10B	72	M	TEST	26	Normal	50	3.042	−1.429	**4.470**
11A	66	F	CONTROL	16	Poor	25	0.958	0.346	**0.612**
11B	65	F	TEST	15	Normal	25	0.650	−0.665	**1.315**
12A	51	F	CONTROL	37	Dense	35	0.495	−0.795	**1.289**
12B	50	F	TEST	35	Normal	30	0.058	−1.713	**1.771**
13A	32	M	CONTROL	26	Poor	40	1.519	−2.259	**3.777**
13B	31	M	TEST	16	Normal	35	1.466	−1.576	**3.041**

**Table 2:** Marginal Bone Loss related to sex and location (mean ± SD, mm) of the implants, in the test and control groups and the cumulative value of all the implants inserted in the study.

**Categories (Test Group)**		**T0**	**T1**	**T1** **-** **T0**
Sex	Male	1.854 ± 0.700	−0.717 ± 0.78	2.571 ± 1.243
	Female	1.452 ± 1.32	−0.76 ± 0.66	2.22 ± 0.92
Location	Posterior maxilla	1.861 ± 0.94	−0.816 ± 0.748	2.678 ± 1.166
	Posterior mandible	1.44 ± 1.007	−0.61 ± 0.703	2.05 ± 0.98
**Categories (Control Group)**		T0	T1	T1-T0
Sex	Male	1.239 ± 0.661	−0.793 ± 0.733	2.032 ± 1.054
	Female	1.705 ± 0.977	−0.043 ± 1.082	1.749 ± 1.060
Location	Posterior maxilla	1.659 ± 0,545	−0.676 ± 0.827	2.335 ± 1.025
	Posterior mandible	1.032 ± 1.031	−0.232 ± 1.092	1.264 ± 0.638
**Categories (All Inserted Implant** **s** **)**		T0	T1	T1-T0
Sex	Male	1.546 ± 0.73	−0.755 ± 0.73	2.30 ± 1.147
	Female	1.578 ± 1.10	−0.40 ± 0.93	1.984 ± 0.968
Location	Posterior maxilla	1.760 ± 0.753	−0.746 ± 0.7655	2.506 ± 1.075
	Posterior mandible	1.236 ± 0.984	−0.42 ± 0.888	1.656 ± 0.883

**Table 3:** The prosthetic complications in both groups.

Groups-	Abutment Decementation	Temporary Crown Decementation	Crown Decementation	Ceramic Chipping
TEST	0	0	0	3 (23.07%)
CONTROL	0	1 (7.69%)	0	2 (15.38%)
TOTAL	0	1 (3.84%)	0	5 (19.23%)
